# Poster 1013: IL-4R alpha antibody inhibits IgE production and airway remodeling in mouse model of house dust mite-induced eosinophilic asthma

**DOI:** 10.1186/1939-4551-7-S1-P8

**Published:** 2014-02-03

**Authors:** Ludmila Kelly, Xia Liu

**Affiliations:** 1 Regeneron Pharmaceuticals, Inc., Tarrytown, NY, USA

## Background

Allergic asthma is associated with T helper 2 (Th2) immune response to an allergen. IL-4 and IL-13, key cytokines involved in Th2 immunity, promote eosinophilic inflammation and B cell isotype switching to IgE. House Dust Mite (HDM) allergen has been shown to induce allergic asthma in a mouse model via Th2 immune response that drives eosinophilia and influx of effector T cells into lung. An IL-4Raplha antibody that blocks signaling of IL-4 and IL-13 through both Type I (IL-4Ralpha/IL-2Rgamma) and Type II (IL-4Ralpha/IL-13R) receptors, and an IL-13Rα2-Fc fusion protein that prevents binding and signaling of IL-13 via Type II receptor, were both tested in the HDM-induced mouse model of asthma.

## Methods

Mice were sensitized daily for 10 days with HDM, rested for 2 weeks, followed by challenge with HDM three times weekly for 8 weeks. Both anti-mouse IL-4Ralpha; antibody (Ab) and fully human anti-human IL-4Raplha Ab (dupilumab) were tested in wild type mice and mice humanized for IL-4 and IL-4Ralpha, respectively. Mice were treated with 50 mg/kg of either Ab twice weekly, starting at week 7 of the experiment. A separate group received mouse IL-13Raplha2-Fc protein using the same dose and regimen. At the end of the experiment, cells obtained by left lung lobe tissue digest were analyzed by flow cytometry. Lung collagen indicative of an airway remodeling was quantified using a Sircol Dye assay, and cardiac lung lobe was used for microarray analysis of gene signature. Levels of HDM-specific IgG1 and total IgE in serum were analyzed by ELISA.

## Results

HDM sensitization and challenge resulted in increased levels of IgE and HDM-specific IgG1. IgE increase was blocked by IL-4Rα Ab but not by IL-13Ralpha2-Fc treatment; HDM-specific IgG1 levels were not affected by either treatment. Collagen content in lung of mice treated with IL-4Ralpha mAb was similar to that observed in mock sensitized and challenged mice. Dupilumab treatment also prevented the influx of eosinophils and inflammatory dendritic cells into lung. HDM-induced changes in gene expression were largely normalized upon dupilumab treatment, as compared to mock treated and sensitized mice.

## Conclusions

Our results demonstrate that blockade of IL-4 signaling via Type I and Type II receptors by IL-4Ralpha antibody suppresses inflammatory and fibrotic changes in lungs of HDM-challenged mice as well as gene signature changes driven by HDM.

